# RETRACTION: Surgical Site Wound Infection and Wound Pain after Video‐Assisted Thoracoscopy in Patients With Lung Cancer: A Meta‐Analysis

**DOI:** 10.1111/iwj.70299

**Published:** 2025-03-06

## Abstract

**RETRACTION:** ZhouJ. , RenZ. , GaoX. , and ZhouX. , “Surgical Site Wound Infection and Wound Pain after Video‐Assisted Thoracoscopy in Patients with Lung Cancer: A Meta‐Analysis,” International Wound Journal 20, no. 9 (2023): 3898–3905, 10.1111/iwj.14237.37293742 PMC10588326

The above article, published online on 09 June 2023, in Wiley Online Library (http://onlinelibrary.wiley.com/), has been retracted by agreement between the journal Editor in Chief, Professor Keith Harding; and John Wiley & Sons Ltd. Following an investigation by the publisher, all parties have concluded that this article was accepted solely on the basis of a compromised peer review process. The editors have therefore decided to retract the article. The authors did not respond to our notice regarding the retraction.

**RETRACTION:** J. Zhou , Z. Ren , X. Gao , and X. Zhou , “Surgical Site Wound Infection and Wound Pain after Video‐Assisted Thoracoscopy in Patients with Lung Cancer: A Meta‐Analysis,” International Wound Journal 20, no. 9 (2023): 3898–3905, 10.1111/iwj.14237.37293742 PMC10588326

The above article, published online on 09 June 2023, in Wiley Online Library (http://onlinelibrary.wiley.com/), has been retracted by agreement between the journal Editor in Chief, Professor Keith Harding; and John Wiley & Sons Ltd. Following an investigation by the publisher, all parties have concluded that this article was accepted solely on the basis of a compromised peer review process. The editors have therefore decided to retract the article. The authors did not respond to our notice regarding the retraction.

